# Differences in judgments on the importance of performance psychological factors among Korean and Chinese football experts

**DOI:** 10.3389/fpsyg.2025.1659102

**Published:** 2025-10-06

**Authors:** Ji-hun Kang, Hong-Fu Tang

**Affiliations:** ^1^Korea National Sport University, Seoul, Republic of Korea; ^2^Shandong First Medical University, Jinan, China

**Keywords:** football, performance psychological factors, cross-culture psychology, analytic hierarchy process, Korea and China

## Abstract

**Purpose:**

This study aimed to assess the importance of performance psychological factors (PPF) among Korean and Chinese football experts (FE) and to identify differences in their judgments regarding PPF.

**Methods:**

In this study, the analytic hierarchy process (AHP) was conducted with 60 participants, including Ph.D. holders in sports psychology with player experience and football coaches with over 10 years of combined experience as players, coaches, and researchers. The standardized scores from the AHP results were used to determine the differences in judgments regarding the importance of PPF between the two countries.

**Results:**

First, Korean FE make judgments on the importance of PPF in the order of factors such as game intelligence and fundamentals, and in the subfactors, practical intelligence, anxiety control, and confidence. Second, Chinese FE make judgments on the importance of PPF in the order of factors such as fundamentals and game intelligence, and in the subfactors, practical intelligence, confidence, and fighting spirit. Third, PPF judged by Korean FE range from confidence (0.050), which is the closest, to pressure control (0.792), which is the farthest. PPF judged by Chinese FE range from analyzing abilities (0.106), the closest, to fighting spirit (0.919), the farthest.

**Conclusions:**

The differences in judgments regarding the importance of PPF in Korean and Chinese FE may reflect varying evaluations of psychological factors influenced by each country's football culture. Overall, this study can serve as a resource for mutual understanding and communication between the two countries.

## 1 Introduction

Korea, China, and Japan in Northeast Asia share harmony and competition in sports. Although China entered sports later than Korea and Japan, it ranks higher in the overall medal standings at the Olympics and Asian Games. However, in football, the most popular sport in the world, the performance of China and Korea differs significantly. Chinese football has made only one World Cup finals appearance and has a 32-year winless record against Korean teams, a situation sometimes referred to as the “Konghanzheng,” which sharply contrasts with the success of Korean football, including 11 World Cup appearances and a semifinal finish. Then, while the two countries share similar cultures, where do their respective differences emerge?

The evaluation of factors that influence a player's performance is affected by culture. Similarities and differences between countries in value judgments (Liang et al., 2024) reflect that evaluation or judgment tendencies may vary depending on environmental contexts. In addition, behaviors of players are influenced by their national cultures, and in fact, playing styles of Korean and Chinese football teams reflect the respective styles of each country. This suggests that culture may influence not only performance but also judgments on performance psychological factors (PPF) in sports. Understanding the importance of PPF in football players as judged in Korean and Chinese contexts enables us to understand each respective culture.

Cross-cultural psychology compares psychological and behavioral similarities and differences across cultures (Yu, 2011). It analyzes behavioral differences based on cultural background (Berry, 1992) and explores the relationship between individual behavior and culture (Berry, 2002). In particular, cross-cultural psychology discusses psychological traits based on culture (Yao and Huang, 2006). From this cross-cultural psychology perspective, Koreans and Chinese share geographical proximity and a Confucianism-centered collectivist culture (Hofstede, 2001). Furthermore, Korean and Chinese cultures may reflect both similarities and differences in understanding.

Sport psychology also emphasizes understanding similarities and differences across cultures. While the ways of understanding emotions may be similar across countries, facial expressions used to express emotions differ (Cowen et al., 2024). Cultural differences may also be reflected in the degree to which students adapt during participation in sports programs (Carter-Thuillier et al., 2023). Furthermore, while emotional intelligence and motivation generally correlate with physical activity across cultures, specific subdomains may give rise to differences (Rogaleva et al., 2024). The need for cultural understanding in psychological support for players has also been suggested (Quartiroli et al., 2023).

The psychology of Korean and Chinese players reflects the characteristics of their respective countries. In fact, football players and coaches observe both similarities and differences in their judgments regarding the importance of PPF (Lee, 2021). Moreover, although both countries show similar attribution styles toward opposing players after matches, Korean players tend to exhibit group-centered thinking, while Chinese players demonstrate individual-centered thinking (Seo and Hwang, 2018). In post-competition self-evaluation among Korean and Chinese swimmers, both groups show similar self-assessment patterns, but Korean players show a higher tendency for social comparison than Chinese players (Hwang et al., 2017). In terms of goal orientation, both countries exhibit high task orientation, but achievement motivation in Korean players tends to be more comparison-focused (Wang and Kim, 2020).

Meanwhile, in football, team styles often mirror national cultures. The mental toughness and tactical understanding of Korean teams, the finesse of Japanese teams, and Spain's tiki-taka style reflect how cultural tendencies influence performance (Hong and Shon, 2010). Specifically, the core determinants of football performance—physical strength, technique, tactics, and psychology—are implemented in ways that best suit the conditions of each country (Yun and Lee, 2006). Such football performance is not merely the sum of individual players' abilities but rather emerges from the synergy of team members, shaping the team's overall playing patterns (Yun and Kim, 2017). In this way, players' adaptation in the football environment is realized through performance.

The PPF framework is structured into four main factors and 14 sub-factors that collectively determine football performance (Yun et al., 2021). Specifically, the PPF framework comprises fundamentals, game intelligence, emotional control, and communication capacity. These PPF components support psychological foundation resources, in-game decision-making and judgment, strategies for maintaining emotional stability, and communication between oneself and others. Specifically, fundamentals refer to psychological foundation resources (Durand-Bush et al., 2023) and include confidence, concentration, fighting spirit, and willpower. Game intelligence consists of resources used for decision-making and judgment during the game (Godbout and Gréhaigne, 2021) and includes practical intelligence, learning capabilities, creativity, and analyzing abilities. Emotional control refers to strategies for maintaining psychological stability (Kang and Yun, 2024), including anxiety control, pressure control, and burden control. Furthermore, communication capacity refers to resources for interacting with oneself and others (Yun et al., 2021) and includes condition, communication with coaches, and communication with teammates. Thus, PPF are structured as a set of factors that influence players' performance.

PPF show both similarities and differences across cultures. Specifically, fundamentals appear to be consistent regardless of cultural background, whereas components such as emotional control and communication capacity may vary depending on culture, sport, and contextual background (Durand-Bush et al., 2023; Yun et al., 2021; Tang, 2024). Additionally, a player's psychological state may change according to the environment and context (Selmi et al., 2023), and judgment tendencies may shift depending on situational factors (Alt et al., 2021). For example, a team's passing network or the verbal and nonverbal communication between teammates can vary depending on the opponent (Mclean et al., 2019). Given the variability of PPF across environments, it is necessary to discuss psychological characteristics that emerge from specific cultural or contextual settings.

Thus, interest in cross-cultural psychology and players' thought processes has helped clarify the unique features of each culture. Discussions from a cross-cultural perspective reflect both similarities and differences in Korean and Chinese sports cultures. In football, factors such as physical fitness, technical skills, tactics, and psychology universally influence performance. Considering that each nation's playing styles are shaped by cultural influences, it is likely that psychological factors affecting performance reflect both the overall football culture and national culture. Therefore, understanding the perspectives on PPF in both countries can support a deeper understanding of each nation's football culture.

Meanwhile, player evaluation reflects expert judgment and decision-making. Football experts (FE) consider contextual and situational factors when evaluating and judging players (Kang and Yun, 2023), and their assessments of players' game intelligence are linked to match data such as passes, shots, and goals (Lennartsson et al., 2015; Vestberg et al., 2020). Furthermore, expert judgments on the importance of performance components are used in identifying athletic talent (Nurjaya et al., 2020). These findings indicate that the variables in judgment and decision-making can change based on context, with the importance of certain components shifting due to environmental and situational factors.

The analytic hierarchy process (AHP), a method that quantifies individual judgments, can be used to structure real-world phenomena through a hierarchical model (Saaty, 1977). AHP allows for the identification of relevant components and the aggregation of expert judgments, thereby systematizing evaluation results (Saaty and Bennett, 1977). The AHP has also been introduced as a research method in sport psychology to evaluate and prioritize these psychological factors (Yun and Kim, 2004). AHP has been applied in football to assess players and make selection decisions (Kang and Yun, 2023), to predict Champions League winners (Syaifudin and Puspitaningayu, 2021), and to compare players' physical performance (Nikjo et al., 2015). It has also been used in baseball to predict championship outcomes (Manoj et al., 2018) and evaluate pitcher performance (Teppa-Garran and Fernández-Da Costa, 2024). Thus, AHP serves as a valuable method for comparison, prediction, and making judgments based on quantified data.

In summary, judgments on the importance of PPF in Korean and Chinese football can contribute to a cultural understanding of how they are perceived in each country. Therefore, this study was conducted to identify differences in judgments on the importance of PPF in Korean and Chinese FE. To achieve this objective, first, judgments on the importance of PPF in Korean and Chinese FE were analyzed; second, differences in judgments on the importance of PPF between the two countries were derived. This study's results will provide a foundation for understanding the football cultures of Korea and China by comparing the importance of PPF in each context.

## 2 Methods

### 2.1 Participants

In this study, to derive the importance of PPF in Korean and Chinese contexts, participants were selected from among individuals with playing experiences in Korean and Chinese sports psychology, Ph.D. holders, football players, and football coaches with more than 10 years of combined experience in playing, coaching, and research. Researchers selected 20 participants from each country, meeting the minimum sample size required to examine group differences effectively (Seong, 2019). Table 1 displays the detailed characteristics of the data contributors.

**Table 1:** Participant characteristics.

**Character**	**Nation (** ***N*** **, M/SD)**		
	**Total**	**Korea**	**China**
Sport psychology Ph.D.	16 (18.4/0.9)	6 (24.8/6.7)	10 (14.6/7.9)
Football coaches	44 (22.5/7.8)	24 (21.9/7.0)	20 (22.6/8.7)
Total	60 (21.6/8.4)	30 (22.9/6.9)	30 (19.9/9.3)

### 2.2 Instruments

**Figure 1:**
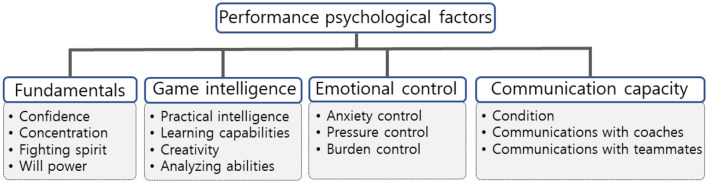
AHP structure (Yun et al., 2021).

**Figure 2:**
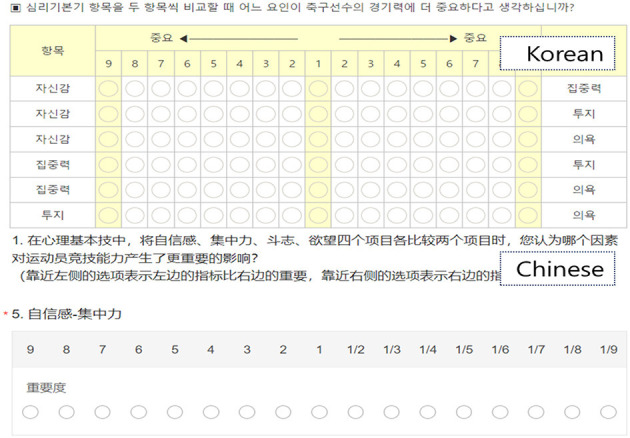
AHP questionnaire.

In this study, data were collected using an AHP questionnaire. The AHP questionnaire was based on the hierarchical structure of PPF affecting football performance presented in Figure 1 (Yun et al., 2021) and was constructed using a 9-point ratio scale based on the structure shown in Figure 1. The Korean AHP questionnaire was developed in an online format (https://www.ssra.or.kr). The Chinese AHP questionnaire was created by translating the Korean AHP questionnaire and was also developed in an online format (https://www.wjx.cn). Figure 2 shows the Korean and Chinese versions of the AHP questionnaire.

### 2.3 Procedure

This study was conducted in three phases: the research design phase, the phase of deriving the importance of PPF in Korean and Chinese, and the phase of deriving variations in judgments on the importance of PPF in Korean and Chinese.

In the research design phase, during the research design phase, the team conducted planning discussions to identify PPF relevant to both Korean and Chinese football contexts (Seong, 2019; Tang, 2024). The research plan also incorporated data comparison methods (Kang and Yun, 2024) to support meaningful cross-national comparisons of the results. The AHP questionnaire included items related to PPF and definitions of each factor. The Chinese AHP questionnaire was developed by translating the Korean AHP questionnaire and term definitions. In the translation process, a researcher with study abroad experience in Korea conducted the first-round translation of items and definitions. The translated questionnaire was reviewed by a Chinese scholar with a Ph.D. in education from Korea, who examined translation accuracy and cultural relevance. Based on this process, the Chinese online AHP questionnaire was finalized.

Data were collected using the Korean and Chinese online AHP questionnaires, and the importance of PPF in Korean and Chinese was calculated. Prior to data collection, institutional review board approval was obtained (R202408200339). Participants who consented to join the study received details about its purpose and confidentiality policy and were instructed to complete the AHP questionnaire through online links sent remotely.

The collected AHP questionnaires were screened using a consistency index threshold of 0.2. The geometric mean of responses was calculated to derive the importance of PPF in Korean and Chinese. Additionally, a correction factor was applied for components with three sub factors by multiplying each sub factor's weight by 0.75 (3/4), in consideration of the number of sub components.

In the phase of deriving variations in judgments on the importance of PPF in Korean and Chinese, the AHP results of Korean and Chinese respondents were converted into standardized scores. To derive the judgment difference values, coordinates were set for the Korean and Chinese axes of PPF, using the standardized scores of each country's general and specific factor weights as axes. The trajectory of points where judgments of Korean and Chinese FE align is represented by the function y = x. The greater the difference in judgments between Korean and Chinese FE, the farther the points deviate from the line y = x. When analyzing inter-country similarities, the closer a data point's distance to the line y = x is to zero, the more aligned the judgments are between the two countries. Conversely, as this distance approaches one, it reflects greater discrepancies in their evaluations. The distance d between the point (a, b) and the line y = x is shown in Equation 1.


(1) $$\begin{array}{c} d=\frac{\left|b-a\right|}{\sqrt{2}} \end{array}$$

The distance d in Equation 1 was computed by inputting the prompt into ChatGPT 4o, and judgment differences between Korean and Chinese FE were calculated by executing the code in Equation 2.


(2) $$\begin{array}{c} d=\left\{vertb-avert\right\}over\left\{sqrt\left\{2\right\}\right\} \end{array}$$

### 2.4 Analysis

In this study, Excel, EC-2000, and ChatGPT 4o were used for data analysis. Excel was used to process collected AHP questionnaire responses (Insert/Function/GEOMEAN) and to calculate standardized scores for differences in judgments on the importance of PPF in Korean and Chinese. EC-2000 was used to perform AHP for calculating the importance of PPF. ChatGPT 4o was utilized to calculate the distance d between points (a, b) using the code provided in Equation 2.

## 3 Results

### 3.1 Judgments on importance of PPF in Korean and Chinese FE

The results of judgments on the importance of PPF in Korean and Chinese FE are as follows. Table 2 presents the importance of PPF as judged by Korean FE.

**Table 2:** Judgments of importance in Korean experts.

**Decisive factors**	**Sub factors**	**Weight**			**Rank**
		**Sub**	**Total**	**Adj**	
Game intelligence^*^(0.351)	Practical intelligence	0.427	0.150	0.150	1
	Learning capabilities	0.225	0.079	0.079	4
	Creativity	0.175	0.061	0.061	5
	Analyzing abilities	0.173	0.061	0.061	6
Fundamentals^*^(0.302)	Confidence	0.392	0.119	0.119	2
	Concentration	0.265	0.080	0.080	3
	Fighting spirit	0.191	0.058	0.058	7
	Will power	0.152	0.046	0.046	11
Emotional control^**^(0.201)	Pressure control	0.359	0.072	0.054	8
	Anxiety control	0.355	0.071	0.053	9
	Burden control	0.286	0.057	0.043	12
Communication capacity^**^(0.146)	Condition	0.465	0.068	0.051	10
	Communications with teammates	0.280	0.041	0.031	13
	Communications with coaches	0.255	0.037	0.028	14

^*^CI = 0.01, ^**^CI = 0.00.

Korean FE judged the importance of PPF in the following order for the general factors: game intelligence, fundamentals, emotional control, and communication capacity. For the specific sub factors, the importance was judged in the following order: practical intelligence, confidence, concentration, learning capabilities, creativity, analyzing abilities, fighting spirit, pressure control, anxiety control, condition, willpower, burden control, communication with teammates, and communication with coaches. Additionally, they considered game intelligence to be 2.4 times more important than communication capacity (0.351/0.146) and practical intelligence to be 5.35 times more important than communication with coaches (0.150/0.028). Table 3 displays the results of Chinese FE' PPF importance judgments.

**Table 3:** Judgments of importance in Chinese experts.

**Decisive factors**	**Sub factors**	**Weight**			**Rank**
		**Sub**	**Total**	**Adj**	
Fundamentals^*^ (0.328)	Confidence	0.316	0.104	0.104	2
	Fighting spirit	0.267	0.088	0.088	3
	Will power	0.234	0.077	0.077	4
	Concentration	0.184	0.060	0.060	9
Game intelligence^*^ (0.321)	Practical intelligence	0.362	0.116	0.116	1
	Creativity	0.231	0.074	0.074	5
	Analyzing abilities	0.208	0.067	0.067	7
	Learning capabilities	0.200	0.064	0.064	8
Communication capacity^*^ (0.193)	Condition	0.501	0.097	0.073	6
	Communications with teammates	0.260	0.050	0.038	11
	Communications with coaches	0.239	0.046	0.035	14
Emotional control^**^ (0.157)	Anxiety control	0.378	0.059	0.044	10
	Pressure control	0.311	0.049	0.037	12
	Burden control	0.311	0.049	0.037	12

^*^CI = 0.01, ^**^CI = 0.00.

Table 3 presents the importance of PPF as judged by Chinese FE.

Chinese FE judged the importance of PPF in the following order for the general factors: fundamentals, game intelligence, communication capacity, and emotional control. For the specific sub factors, the importance was judged in the following order: practical intelligence, confidence, fighting spirit, willpower, creativity, condition, analyzing abilities, learning capabilities, concentration, anxiety control, communication with teammates, pressure control, and communication with coaches. Furthermore, they considered game intelligence to be 2.09 times more important than emotional control (0.328/0.157) and practical intelligence to be 3.31 times more important than communication with coaches (0.116/0.035).

### 3.2 Differences in judgments on importance of PPF in Korean and Chinese FE

**Figure 3:**
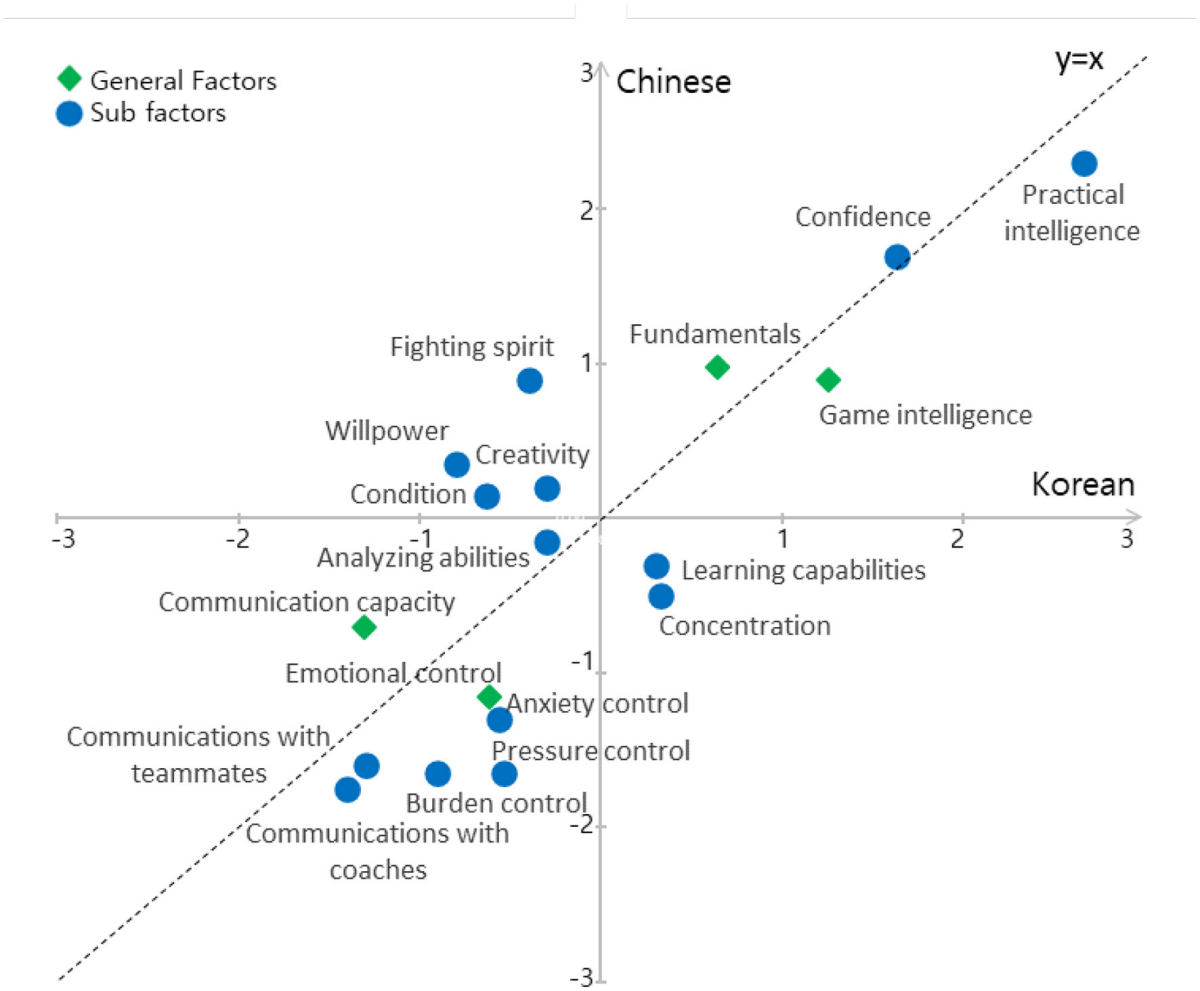
Difference for judgments of importance.

Differences in judgments on the importance of PPF in Korean and Chinese FE were examined through a statistical process by converting the importance weights of PPF judgments into standardized scores. Additionally, judgment differences on the importance of PPF in Korean and Chinese FE can be summarized as follows: when judgments of Korean and Chinese experts are plotted on separate axes and importance scores are represented as coordinate points, complete agreement between Korean and Chinese FE is indicated by the line y = x. Accordingly, standardized score coordinates x, y for differences in PPF importance judgments are presented in Figure 3.

The judgment differences between Korean and Chinese FE are illustrated in Figure 3, and the distance d between each importance judgment coordinate and the line y = x are shown in Table 4.

**Table 4:** Response differences distance from function y = x.

**Category**	**Highly valued by Korean (d)**	**Highly valued by Chinese (d)**
General factors	Game intelligence (0.262), Emotional control (0.389)	Fundamentals (0.233), communication capacity (0.417)
Sub factors	Confidence (0.050), practical intelligence (0.262), learning capabilities (0.424), anxiety control (0.516), burden control (0.530), concentration (0.587), pressure control (0.792)	communications with teammates (0.212), communications with coaches (0.248), analyzing abilities (0.106), creativity (0.354), condition (0.552), will power (0.918), fighting spirit (0.919)

Differences in judgments on the importance of PPF in Korean and Chinese FE can be categorized into results where Korean FE rated the importance higher and results where Chinese FE rated the importance higher. As shown in Table 4, when considering the distance from the line y = x, PPF judgments by Korean FE range from confidence, with the smallest distance (0.050), to pressure control, with the largest distance (0.792). PPF judgments by Chinese FE range from analyzing abilities with the smallest distance (0.106) to fighting spirit with the largest distance (0.919). The distance from the line y = x represents the magnitude of differences in judgments on the importance between Korean and Chinese FE.

## 4 Discussion

### 4.1 Judgments on importance of PPF in Korean and Chinese FE

Korean FE judged the importance of PPF in the following order for the general factors: game intelligence, fundamentals, emotional control, and communication capacity. For the specific sub factors, the importance was ranked in the order of practical intelligence, confidence, concentration, learning capabilities, and so on. Game intelligence, practical intelligence, confidence, concentration, and learning capabilities were closely related to performance. PPF of players emphasize decision-making and concentration (Beńıtez-Sillero et al., 2021; Simonenkova and Sopov, 2020), and the relationship between performance and factors such as confidence, concentration, and cognitive ability (Abdullah et al., 2016) is well established. Players with high concentration and game intelligence tend to achieve better match performance (Németh and Balogh, 2021). In practice, Korean football coaches frequently give instructions to players during matches to “have confidence” or “focus more.” This suggests that Korean FE place importance on players being in a psychological state that allows them to adapt to the game while fully demonstrating their own performance.

Chinese FE judged the importance of PPF in the following order for the general factors: fundamentals, game intelligence, communication capacity, and emotional control. For the specific sub factors, practical intelligence, confidence, fighting spirit, and willpower were ranked as most important. Confidence (Durand-Bush et al., 2023) and practical intelligence (Godbout and Gréhaigne, 2021) are PPF that are emphasized in football. In addition, mental strength and fighting spirit are particularly emphasized in Chinese football (Lee, 2021). The importance of fighting spirit and willpower in football continues to be recognized (Yun, 2004; Yun et al., 2021). These findings indicate that Chinese FE consider the player's effort to invest their maximum internal resources into the match as highly important.

### 4.2 Differences in judgments on importance of PPF in Korean and Chinese FE

Differences in judgments on the importance of PPF in Korean and Chinese FE were compared. Furthermore, judgments on the importance of PPF in Korean and Chinese contexts reflect the respective football cultures of each country. In Table 4, the distance d greater than 0.500 indicates that the coordinate points lie relatively far from the line y = x shown in Figure 1. Based on this, items where Korean FE showed significant differences from Chinese FE included anxiety control (0.516), burden control (0.530), concentration (0.587), and pressure control (0.792). Emotional control is not only a key resource influencing player performance (Durand-Bush et al., 2023) but also reflects the Korean football culture that emphasizes maintaining a psychologically stable state (Yun et al., 2021). This tendency is also seen in elite Korean players such as Heung-min Son and Kang-in Lee, who demonstrated psychological stability in high-pressure situations, particularly in European leagues.

In contrast, items where Chinese FE showed significant differences from Korean FE included condition (0.552), willpower (0.918), and fighting spirit (0.919). Condition relates to self-awareness and self-regulation, aligning more with individual-centered thinking rather than communication or collaboration. This reflects the characteristics of Chinese players who prioritize their performance (Seo and Hwang, 2018), as well as cultural tendencies within the Chinese sports system. In practice, the Chinese football sector receives support through government-led policies, and the emphasis on fighting spirit and mental toughness reflects elements of Chinese football culture (Lee, 2021).

In summary, the PPF judgments of Korean and Chinese football experts reflect both the shared Confucian cultural heritage of Korea and China and the unique socio-cultural environments of each country. Both nations are deeply rooted in collectivist cultures shaped by Confucian values (Hofstede, 2001). However, while their value assessments exhibit similarities, distinct differences across countries (Liang et al., 2024) underscore the significance of environmental context in the judgment process. Within this framework, analyzing individual and group behavior from a cross-cultural perspective (Berry, 2002) can enhance mutual understanding and communication.

In this way, differences in PPF judgments between Korean and Chinese FE may reflect culturally shaped psychological evaluations, influenced by each country's unique football culture.

## 5 Limitations and future research

Based on the process and findings of this study, the following suggestions are proposed for future research:

First, a nuanced understanding of Korean and Chinese football cultures is essential. This study explored the importance of PPF through the lens of experts from each country, considering their cultural perspectives. Future research should examine how each country evaluates the other and how these judgments evolve over time. Recognizing shifting perspectives and trends between Korea and China will further enrich the ongoing discourse on PPF.

Second,it is necessary to explore the importance of PPF from the perspectives of Korean and Chinese football players. This study focused on the judgments of FE regarding the importance of PPF. Considering the results, it is likely that there may be differences in how Korean and Chinese football players perceive the importance of PPF. Investigating the judgments of players from both countries can enrich the discourse on PPF within the football community. Furthermore, given the increasing trend of Korean coaches advancing into Chinese football, understanding players' perceptions on the importance of PPF can serve as a valuable tool for Korean coaches working with Chinese players.

Third, there is a need to expand the scope of research on the importance of judgments on PPF to other sports in Korea and China. In reality, China is particularly strong in sports such as table tennis, gymnastics, and skating. Expanding to these sports offers insights into the differing valuations of PPF in areas where China excels compared to its weaker sports. Such an expansion would offer a more comprehensive understanding of PPF importance across different contexts in both countries. Research that evolves from existing studies can contribute not only to academic understanding but also serve as a foundational reference for communication between the sports communities and scholars of both nations.

Forth, the results regarding judgments on the importance of PPF in Korean and Chinese football will be used as a reference point for academic and practical exchange between the football communities of both countries. Korea and China share similar yet distinct football cultures, and the differences identified in this study regarding the importance of PPF can serve as a useful basis for understanding each other's football culture. In fact, the Chinese football sector is continuously striving to develop a unique Chinese football culture and improve football performance. Therefore, this study will be used as a resource to foster mutual understanding between the Chinese and Korean football communities.

## 6 Conclusion

The conclusions of this study are as follows:

Korean FE place importance on players' psychological adaptation to the game through emotional stability within the context of PPF. In contrast, Chinese FE emphasize players' attitudes as the psychological foundation of performance. Korean FE rate emotional control highly, while Chinese FE emphasize condition, fighting spirit, and willpower. Furthermore, differences in judgments on the importance of PPF in Korean and Chinese FE reflect how the evaluation of psychological factors can vary depending on each country's football culture.

## Data Availability

The data that support the findings of this study are available from the corresponding author, upon reasonable request.
